# *N*,*N*′-Bis(pyridin-3-yl)terephthalamide–terephthalic acid (1/1)

**DOI:** 10.1107/S1600536811041596

**Published:** 2011-10-29

**Authors:** Ji-lin Lu, Xue-wen Liu, Lin Li, Yuan-dao Chen, Guang-yu Shen

**Affiliations:** a College of Chemistry and Chemical Engineering, Hunan University of Arts and Science, ChangDe, Hunan provice 415000, People’s Republic of China

## Abstract

In the title compound, C_18_H_14_N_4_O_2_·C_8_H_6_O_4_, both types of mol­ecule lie on inversion centers. In the *N*,*N*′-bis­(pyridin-3-yl)terephthalamide mol­ecule, the pyridine ring forms a dihedral angle of 11.33 (9)° with the central benzene ring. In the crystal, N—H⋯O and O—H⋯N hydrogen bonds connect the components into a three-dimensional network.

## Related literature

For related structures, see: Xiao *et al.* (2011 ▶), Wang *et al.* (2009 ▶). 
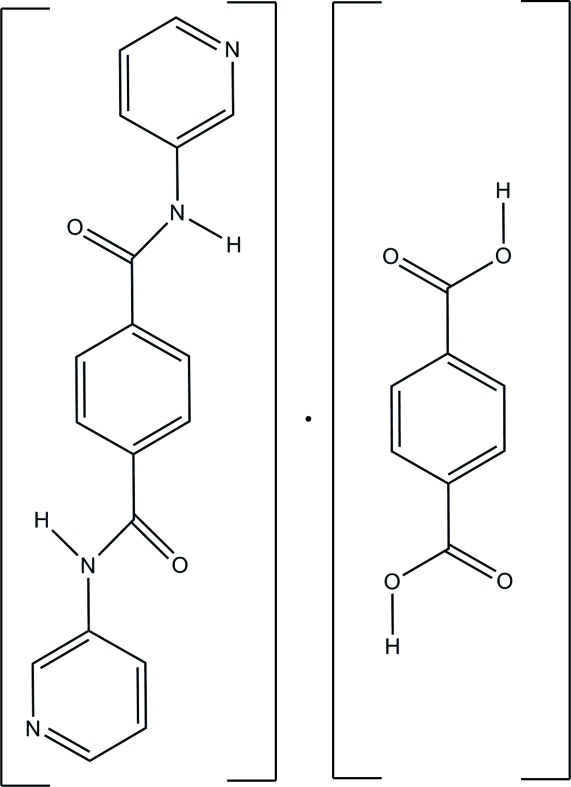


## Experimental

### 

#### Crystal data

C_18_H_14_N_4_O_2_·C_8_H_6_O_4_
*M* *_r_* = 484.46
Monoclinic, 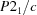

*a* = 11.0001 (3) Å
*b* = 10.8080 (2) Å
*c* = 9.6903 (2) Å
β = 106.830 (2)°
*V* = 1102.73 (4) Å^3^
*Z* = 2
Mo *K*α radiation
μ = 0.11 mm^−1^
*T* = 296 K
0.25 × 0.24 × 0.22 mm

#### Data collection

Bruker SMART CCD diffractometer
Absorption correction: multi-scan (SABADS; Sheldrick, 1996) ▶ *T*_min_ = 0.974, *T*_max_ = 0.977
8117 measured reflections
1939 independent reflections
1640 reflections with *I* > 2σ(*I*)
*R*_int_ = 0.035

#### Refinement

*R*[*F* ^2^ > 2σ(*F* ^2^)] = 0.040
*wR*(*F* ^2^) = 0.095
*S* = 1.07
1939 reflections
171 parameters
H atoms treated by a mixture of independent and constrained refinement
Δρ_max_ = 0.14 e Å^−3^
Δρ_min_ = −0.19 e Å^−3^

### 

Data collection: *SMART* (Bruker, 1998 ▶); cell refinement: *SAINT* (Bruker, 1998 ▶); data reduction: *SAINT*; program(s) used to solve structure: *SHELXS97* (Sheldrick, 2008 ▶); program(s) used to refine structure: *SHELXL97* (Sheldrick, 2008 ▶); molecular graphics: *DIAMOND* (Brandenburg, 1999 ▶); software used to prepare material for publication: *SHELXTL* (Sheldrick, 2008 ▶).

## Supplementary Material

Crystal structure: contains datablock(s) global, I. DOI: 10.1107/S1600536811041596/lh5338sup1.cif

Structure factors: contains datablock(s) I. DOI: 10.1107/S1600536811041596/lh5338Isup2.hkl

Supplementary material file. DOI: 10.1107/S1600536811041596/lh5338Isup3.cml

Additional supplementary materials:  crystallographic information; 3D view; checkCIF report

## Figures and Tables

**Table 1:** Hydrogen-bond geometry (Å, °)

*D*—H⋯*A*	*D*—H	H⋯*A*	*D*⋯*A*	*D*—H⋯*A*
N2—H2*N*⋯O1^i^	0.89 (2)	1.98 (2)	2.8616 (18)	171.3 (18)
O2—H1*N*⋯N1^ii^	1.00 (3)	1.69 (3)	2.6938 (19)	178 (2)

Symmetry codes: (i) 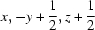; (ii) 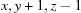.

**Fig. 1.**
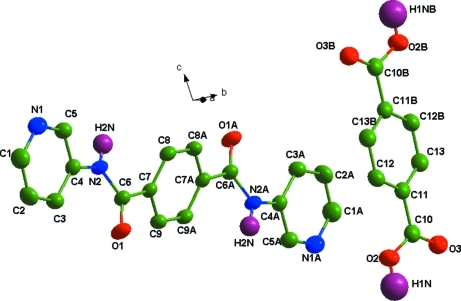
The molecular structure of the title compound with displacement ellipsoids drawn at the 50% probability level (symmetry code; (A) -x + 1, -y + 1, -z + 1; (B) -x + 2, -y + 2, -z + 1). Hydrogen atoms bonded to C atoms are not shown.

**Fig. 2.**
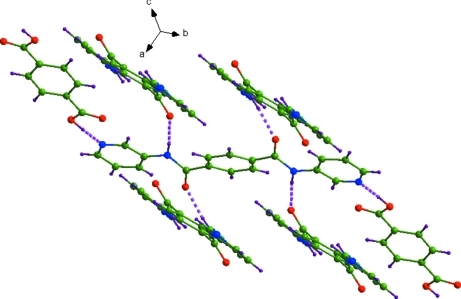
Part of the crystal structure with hydrogen bonds shown as pink dashed lines. H atoms are purple.

## supplementary crystallographic information

### Comment

Pyridine amide derivatives and carboxylic acids easily form hydrogen bonds therefore they are useful to construct supramoleculur structures (e.g. Xiao *et al.*, 2011; Wang *et al.*, 2009). Herein, we use N,N'-di(pyridin-3-yl)terephthalamide and terephthalic acid to construct a supramolecular compound. The crystal structure of the title compound is presented herein.

The molecular structure of the title compound is shown in Fig. 1. The symmetry unique pyridine ring forms a dihedral angle of 11.33 (9)° with the central benzene ring. In the crystal, N—H···O and O—H···N hydrogen bonds connect the components of the structure into a three dimensional network (Fig. 2).

### Experimental

N,N'-di(pyridin-3-yl)terephthalamide (0.2 mmol) and terephthalic acid (0.2 mmol) was sealed in a teflon reactor with 6 mL water, and heated at 433 K for 2 days, and then cooled to room temperature. The single crystals were obtained by slow evaporation.

### Refinement

H atoms bonded to C atoms were placed in calculated positions with C—H = 0.93Å and included using a riding-model approximation with *U*_iso_(H) = 1.2*U*_eq_(C). H atoms bonded to O and N atoms were refined independently with isotropic displacement parameters.

### Crystal data

**Table:**

C_18_H_14_N_4_O_2_·C_8_H_6_O_4_	*F*(000) = 504
*M**_r_* = 484.46	*D*_x_ = 1.459 Mg m^−^^3^
Monoclinic, *P*2_1_/*c*	Mo *K*α radiation, λ = 0.71073 Å
Hall symbol: -P 2ybc	Cell parameters from 2279 reflections
*a* = 11.0001 (3) Å	θ = 2.7–25.2°
*b* = 10.8080 (2) Å	µ = 0.11 mm^−^^1^
*c* = 9.6903 (2) Å	*T* = 296 K
β = 106.830 (2)°	Block, colourless
*V* = 1102.73 (4) Å^3^	0.25 × 0.24 × 0.22 mm
*Z* = 2	

### Data collection

**Table:**

Bruker SMART CCD diffractometer	1939 independent reflections
Radiation source: fine-focus sealed tube	1640 reflections with *I* > 2σ(*I*)
graphite	*R*_int_ = 0.035
φ and ω scans	θ_max_ = 25.0°, θ_min_ = 1.9°
Absorption correction: multi-scan (SABADS; Sheldrick, 1996)	*h* = −13→12
*T*_min_ = 0.974, *T*_max_ = 0.977	*k* = −12→11
8117 measured reflections	*l* = −11→11

### Refinement

**Table:**

Refinement on *F*^2^	Primary atom site location: structure-invariant direct methods
Least-squares matrix: full	Secondary atom site location: difference Fourier map
*R*[*F*^2^ > 2σ(*F*^2^)] = 0.040	Hydrogen site location: inferred from neighbouring sites
*wR*(*F*^2^) = 0.095	H atoms treated by a mixture of independent and constrained refinement
*S* = 1.07	*w* = 1/[σ^2^(*F*_o_^2^) + (0.0378*P*)^2^ + 0.3019*P*] where *P* = (*F*_o_^2^ + 2*F*_c_^2^)/3
1939 reflections	(Δ/σ)_max_ < 0.001
171 parameters	Δρ_max_ = 0.14 e Å^−^^3^
0 restraints	Δρ_min_ = −0.19 e Å^−^^3^

### Special details

**Table:**

Geometry. All e.s.d.'s (except the e.s.d. in the dihedral angle between two l.s. planes) are estimated using the full covariance matrix. The cell e.s.d.'s are taken into account individually in the estimation of e.s.d.'s in distances, angles and torsion angles; correlations between e.s.d.'s in cell parameters are only used when they are defined by crystal symmetry. An approximate (isotropic) treatment of cell e.s.d.'s is used for estimating e.s.d.'s involving l.s. planes.
Refinement. Refinement of *F*^2^ against ALL reflections. The weighted *R*-factor *wR* and goodness of fit *S* are based on *F*^2^, conventional *R*-factors *R* are based on *F*, with *F* set to zero for negative *F*^2^. The threshold expression of *F*^2^ > σ(*F*^2^) is used only for calculating *R*-factors(gt) *etc*. and is not relevant to the choice of reflections for refinement. *R*-factors based on *F*^2^ are statistically about twice as large as those based on *F*, and *R*- factors based on ALL data will be even larger.

### Fractional atomic coordinates and isotropic or equivalent isotropic displacement parameters (Å^2^)

**Table:**

	*x*	*y*	*z*	*U*_iso_*/*U*_eq_	
C10	0.91350 (16)	1.01651 (17)	0.18960 (19)	0.0385 (4)	
C11	0.95943 (16)	1.00643 (16)	0.35009 (18)	0.0358 (4)	
C8	0.58897 (16)	0.45790 (15)	0.62372 (17)	0.0336 (4)	
H8	0.6489	0.4302	0.7068	0.040*	
C7	0.54563 (16)	0.37897 (14)	0.50665 (16)	0.0308 (4)	
C5	0.74809 (16)	0.04149 (16)	0.79727 (18)	0.0367 (4)	
H5	0.7580	0.1028	0.8671	0.044*	
C3	0.66694 (17)	−0.02113 (16)	0.55212 (18)	0.0381 (4)	
H3	0.6219	−0.0058	0.4565	0.046*	
C6	0.59441 (16)	0.25022 (15)	0.50444 (17)	0.0339 (4)	
C9	0.45681 (17)	0.42247 (15)	0.38296 (17)	0.0351 (4)	
H9	0.4279	0.3704	0.3038	0.042*	
C12	1.01793 (16)	0.89971 (16)	0.41856 (19)	0.0388 (4)	
H12	1.0304	0.8326	0.3642	0.047*	
C4	0.68076 (15)	0.06989 (15)	0.65646 (17)	0.0313 (4)	
C13	0.94247 (17)	1.10642 (16)	0.43264 (19)	0.0399 (4)	
H13	0.9041	1.1782	0.3874	0.048*	
C2	0.72163 (18)	−0.13483 (16)	0.5939 (2)	0.0429 (5)	
H2	0.7154	−0.1971	0.5259	0.051*	
C1	0.78552 (18)	−0.15624 (17)	0.7362 (2)	0.0460 (5)	
H1	0.8204	−0.2341	0.7630	0.055*	
N1	0.79926 (14)	−0.06916 (14)	0.83764 (15)	0.0423 (4)	
N2	0.62725 (14)	0.18931 (13)	0.63090 (14)	0.0338 (3)	
H2N	0.6221 (18)	0.2299 (18)	0.709 (2)	0.048 (5)*	
O1	0.60228 (13)	0.20383 (11)	0.39151 (12)	0.0476 (4)	
O2	0.91449 (14)	0.91030 (12)	0.12264 (15)	0.0524 (4)	
H1N	0.874 (3)	0.918 (3)	0.016 (3)	0.095 (9)*	
O3	0.87627 (13)	1.11288 (12)	0.12828 (13)	0.0501 (4)	

### Atomic displacement parameters (Å^2^)

**Table:**

	*U*^11^	*U*^22^	*U*^33^	*U*^12^	*U*^13^	*U*^23^
C10	0.0381 (9)	0.0349 (10)	0.0398 (10)	−0.0038 (8)	0.0069 (8)	0.0027 (8)
C11	0.0364 (9)	0.0332 (10)	0.0353 (9)	−0.0037 (7)	0.0063 (7)	0.0034 (7)
C8	0.0435 (9)	0.0310 (9)	0.0255 (8)	0.0035 (7)	0.0086 (7)	0.0045 (7)
C7	0.0437 (9)	0.0256 (9)	0.0255 (8)	0.0017 (7)	0.0136 (7)	0.0033 (7)
C5	0.0458 (10)	0.0313 (10)	0.0321 (9)	0.0041 (8)	0.0100 (8)	0.0008 (7)
C3	0.0485 (10)	0.0328 (10)	0.0317 (9)	0.0024 (8)	0.0094 (8)	−0.0004 (7)
C6	0.0472 (10)	0.0294 (9)	0.0273 (9)	0.0014 (7)	0.0143 (7)	0.0014 (7)
C9	0.0514 (10)	0.0279 (9)	0.0258 (8)	0.0002 (7)	0.0109 (8)	−0.0011 (7)
C12	0.0434 (10)	0.0313 (9)	0.0388 (10)	−0.0002 (8)	0.0072 (8)	−0.0022 (8)
C4	0.0384 (9)	0.0259 (9)	0.0311 (9)	0.0028 (7)	0.0123 (7)	0.0026 (7)
C13	0.0430 (10)	0.0312 (10)	0.0408 (10)	0.0023 (8)	0.0047 (8)	0.0036 (8)
C2	0.0539 (11)	0.0282 (10)	0.0452 (11)	0.0045 (8)	0.0122 (9)	−0.0059 (8)
C1	0.0530 (11)	0.0311 (10)	0.0509 (12)	0.0092 (8)	0.0105 (9)	0.0034 (9)
N1	0.0493 (9)	0.0362 (9)	0.0377 (8)	0.0086 (7)	0.0066 (7)	0.0049 (7)
N2	0.0533 (9)	0.0253 (8)	0.0241 (7)	0.0066 (6)	0.0132 (6)	0.0012 (6)
O1	0.0845 (10)	0.0333 (7)	0.0306 (7)	0.0124 (6)	0.0255 (6)	0.0033 (5)
O2	0.0773 (10)	0.0365 (8)	0.0357 (8)	0.0039 (7)	0.0044 (7)	0.0003 (6)
O3	0.0656 (9)	0.0373 (8)	0.0401 (7)	0.0025 (6)	0.0037 (6)	0.0063 (6)

### Geometric parameters (Å, °)

**Table:**

C10—O3	1.210 (2)	C6—O1	1.2293 (19)
C10—O2	1.320 (2)	C6—N2	1.345 (2)
C10—C11	1.494 (2)	C9—C8^i^	1.382 (2)
C11—C13	1.389 (2)	C9—H9	0.9300
C11—C12	1.392 (2)	C12—C13^ii^	1.382 (2)
C8—C9^i^	1.382 (2)	C12—H12	0.9300
C8—C7	1.389 (2)	C4—N2	1.410 (2)
C8—H8	0.9300	C13—C12^ii^	1.382 (2)
C7—C9	1.391 (2)	C13—H13	0.9300
C7—C6	1.494 (2)	C2—C1	1.374 (3)
C5—N1	1.331 (2)	C2—H2	0.9300
C5—C4	1.385 (2)	C1—N1	1.337 (2)
C5—H5	0.9300	C1—H1	0.9300
C3—C2	1.377 (2)	N2—H2N	0.89 (2)
C3—C4	1.387 (2)	O2—H1N	1.00 (3)
C3—H3	0.9300		
			
O3—C10—O2	123.83 (16)	C8^i^—C9—H9	119.6
O3—C10—C11	122.57 (16)	C7—C9—H9	119.6
O2—C10—C11	113.58 (15)	C13^ii^—C12—C11	119.97 (16)
C13—C11—C12	119.40 (16)	C13^ii^—C12—H12	120.0
C13—C11—C10	118.80 (15)	C11—C12—H12	120.0
C12—C11—C10	121.80 (16)	C5—C4—C3	118.38 (15)
C9^i^—C8—C7	120.14 (15)	C5—C4—N2	116.91 (15)
C9^i^—C8—H8	119.9	C3—C4—N2	124.68 (15)
C7—C8—H8	119.9	C12^ii^—C13—C11	120.63 (16)
C8—C7—C9	119.05 (15)	C12^ii^—C13—H13	119.7
C8—C7—C6	122.97 (14)	C11—C13—H13	119.7
C9—C7—C6	117.92 (14)	C1—C2—C3	119.90 (17)
N1—C5—C4	123.26 (16)	C1—C2—H2	120.1
N1—C5—H5	118.4	C3—C2—H2	120.1
C4—C5—H5	118.4	N1—C1—C2	122.36 (17)
C2—C3—C4	118.19 (16)	N1—C1—H1	118.8
C2—C3—H3	120.9	C2—C1—H1	118.8
C4—C3—H3	120.9	C5—N1—C1	117.91 (15)
O1—C6—N2	122.81 (16)	C6—N2—C4	126.60 (14)
O1—C6—C7	120.72 (14)	C6—N2—H2N	117.7 (12)
N2—C6—C7	116.47 (14)	C4—N2—H2N	115.4 (12)
C8^i^—C9—C7	120.81 (15)	C10—O2—H1N	111.7 (16)
			
O3—C10—C11—C13	9.3 (3)	N1—C5—C4—C3	−0.6 (3)
O2—C10—C11—C13	−169.05 (16)	N1—C5—C4—N2	177.29 (16)
O3—C10—C11—C12	−171.05 (17)	C2—C3—C4—C5	−0.4 (2)
O2—C10—C11—C12	10.6 (2)	C2—C3—C4—N2	−178.11 (16)
C9^i^—C8—C7—C9	0.6 (3)	C12—C11—C13—C12^ii^	−0.6 (3)
C9^i^—C8—C7—C6	177.70 (15)	C10—C11—C13—C12^ii^	179.14 (16)
C8—C7—C6—O1	−146.83 (17)	C4—C3—C2—C1	1.3 (3)
C9—C7—C6—O1	30.4 (2)	C3—C2—C1—N1	−1.3 (3)
C8—C7—C6—N2	33.8 (2)	C4—C5—N1—C1	0.6 (3)
C9—C7—C6—N2	−149.04 (16)	C2—C1—N1—C5	0.3 (3)
C8—C7—C9—C8^i^	−0.6 (3)	O1—C6—N2—C4	3.8 (3)
C6—C7—C9—C8^i^	−177.85 (15)	C7—C6—N2—C4	−176.86 (15)
C13—C11—C12—C13^ii^	0.6 (3)	C5—C4—N2—C6	157.60 (17)
C10—C11—C12—C13^ii^	−179.13 (16)	C3—C4—N2—C6	−24.7 (3)

Symmetry codes: (i) −*x*+1, −*y*+1, −*z*+1; (ii) −*x*+2, −*y*+2, −*z*+1.

### Hydrogen-bond geometry (Å, °)

**Table:**

*D*—H···*A*	*D*—H	H···*A*	*D*···*A*	*D*—H···*A*
N2—H2N···O1^iii^	0.89 (2)	1.98 (2)	2.8616 (18)	171.3 (18)
O2—H1N···N1^iv^	1.00 (3)	1.69 (3)	2.6938 (19)	178 (2)

Symmetry codes: (iii) *x*, −*y*+1/2, *z*+1/2; (iv) *x*, *y*+1, *z*−1.
